# An Experiment with Kerosolene—A New Anæsthetic

**Published:** 1861-08

**Authors:** E. Andrews

**Affiliations:** Professor of Surgery in Lind University


ARTICLE IV.

### AN EXPERIMENT WITH KEROSOLENE—A NEW ANÆSTHETIC.

By E. ANDREWS, M. D., Prof. of Surgery in Lind University.

This fluid is a hydrocarbon of the series obtained by distilation of bituminous coal. It appears to be the most volatile of its class, having less specific gravity than ether, and seeming to be about equally volatile. The odor is but slight, and in kind resembles that of other coal oils. Dr. Bigelow, of Boston, states that it is "as tasteless as water," but this is an error, as it has a distinct, though not strong, flavor. On the whole it is much less pungent, both to taste and smell, than chloroform and ether.

It has been my opinion for some years that all the volatile hydrocarbons were anæsthetics, including coal gas, which latter I have proved by experiment.

The anæsthetic qualities of kerosolene were first observed in a kerosene factory, where it was noticed that men who were sent down into the vats became narcotized by the inhaled vapor, and on removal to fresh air recovered without injury. As the kerosolene is the most volatile of the compounds present in these oils, it was presumed to be the chief anæsthetic agent present, and was selected for experiment.

On the 19th of July, Dr. Dickinson, of St. Louis, called upon me and presented a can of the fluid, with a request that it might be tested. As there happened to be present at the moment a patient of the Chicago Dispensary, to be sounded for stone in the bladder, I determined to try the article at once. Accordingly, the patient was laid upon a sofa, and at my request, Dr. Dickinson commenced the administration. It was given by sprinkling upon a handkerchief in the same manner as chloroform, taking care to secure an abundant access of air. The patient seemed to take it with ease and comfort, and for several minutes everything went on well. The pulse was full, and somewhat accelerated, but it did not intermit or flag in any part of the procedure. At length the anæsthesia began to come on, when the countenance appeared flushed, and the eye a little unnaturally open and staring. In a short time the patient made an effort to rise up in a sitting posture, and was immediately attacked with moderate convulsions. The administration was discontinued until these effects subsided, and then carefully resumed, but the result was another attack of the convulsions. Again, suspending the inhalation until the muscular excitement ceased, a third effort was made to proceed, but this was also checked by a third attack of the spasms. Great as was my desire to test the article, I did not deem it prudent to persist in the face of such warnings, therefore laying aside the kerosolene, the anæsthesia was finished with a mixture of ether and chloroform. No further convulsive motions occurred. Both the pulse and the respiration were well sustained during the inhalation of the kerosolene, the only exception being that during the convulsions a moderate check of respiratory movement occurred, in consequence of the rigid contraction of the muscles of the chest. The result was a momentary dusky redness of the face at each convulsion, similar to that which occurs in epilepsy.

On the whole, the symptoms were alarming but not disastrous. If this shall be found a frequent result, it will effectually exclude the article from use.

Dr. H. J. Bigelow, of Boston, has experimented once upon himself, and four times upon others, with this fluid. In his own case its action was all that could be desired, but in all the other instances unpleasant effects occurred, such as failure of respiration, intermittent pulse, and muscular rigidity. I make an extract from his communication to the Boston *Medical and Surgical Journal:*

•This fluid presents remarkable properties. It is tasteless as water, volatile and inflammable as ether, though burning with a dense white light; of a faint chloroform odor, which, as it evaporates, changes to that of coal tar, and then disappears absolutely and altogether; so that a handkerchief, saturated with the fluid has, at the end of a few minutes, when dry, no odor at all, nor has the room or atmosphere where it has been used, any trace of its presence. Both ether and chloroform leave, in different degrees, a persistent, *fadé* and stale aroma after evaporation, as is well known. They are also far less agreeable to inhale than this new agent, which has thus an obvious advantage over either of them.

A few whiffs were sufficient assurance of its efficacy as an anæsthetic, which, with its other qualities, as I ventured to remark, would place the kerosolene beyond any known anæsthetic, provided its use was not followed by headache, vertigo or other unpleasant symptoms, and provided it should prove as free from danger as ether.

Subsequently, I inhaled the new vapor, which Dr. Hodges, at my request, administered. Complete insensibility supervened, lasting several minutes, with some diminution of the volume of the pulse. Its effect was wholly agreeable, leaving neither headache nor nausea, nor bad taste.

I have this morning administered it to three surgical patients. The first, a girl of 19, presenting some hysteric tendencies, having thrust some twenty needles in her leg, was wholly insensible during the extraction of four of those which remained. Yet there was more cough than I had expected from the wholly unirritating odor of the vapor, more muscular rigor than usual in favorable anæsthesia, and more intermittence of the pulse.

In a second patient, to whom it was given preparatory to an operation upon the face, insensibility was equally complete. But this woman did not take it kindly, and its complete effect was attended by so feeble and intermittent a pulse as to lead me to desist until she had recovered. A second attempt reproduced, with anæsthesia, the feeble and intermittent pulse, and I again desisted. Upon her recovery, I gave her common ether vapor, which she afterwards said was less agreeable, but which was followed by complete insensibility, the pulse beating steadily and full, at 76. Though this patient perhaps succumbed more readily to a third anæsthesia, there seemed to be in the two first trials a certain degree of purple color and asphyxia, with its attendant spasm, which I have elsewhere described as an occasional and disagreeable symptom of anæsthesia. To guard against this asphyxia, which might possibly have resulted from the folded towel, upon which I habitually administer ether, I tried in the next case an open sponge. The subject required a considerable incision for a mammary abscess, and was a patient of Dr. H. G. Clark, with whose assent I tried the kerosolene. In spite of the open sponge, the symptoms of asphyxia again appeared, suggesting to Dr. Clark before operating, their resemblance to those resulting from charcoal gas. The color was livid, and the rigidity marked. In each of these cases, the quantity used was from one to two ounces.

In conclusion, it may be remarked of these three cases, that they are insufficient for satisfactory demonstration, and that their common and unfavorable symptoms may well have been but a coincidence; yet they suggest some caution in the use of the kerosolene vapor. It is probably more potent than that of ether, requires a free admixture of air, and may produce upon the system some impression or influence, other than that of the mere intoxication attendant upon the use of ether. In awaiting further evidence, it may be considered established that kerosolene is an anæsthetic of undoubted efficiency, and that it possesses certain remarkable and attractive properties peculiar to itself.

